# Cumulative Stress Burden and Association With DNA Methylation in Ethiopian American Immigrants: Protocol for a Community-Engaged, Biopsychosocial Study

**DOI:** 10.2196/85971

**Published:** 2026-02-10

**Authors:** Lara Stone, Lili Ding, Meron Hirpa Kassa, Beakal Amsalu, Bruktawit Getnet, Hermella Eshete, Tesfaye B Mersha, Lisa M Vaughn

**Affiliations:** 1 Division of Emergency Medicine Cincinnati Children's Hospital Medical Center Cincinnati, OH United States; 2 Department of Pediatrics College of Medicine University of Cincinnati Cincinnati, OH United States; 3 Division of Biostatistics/Epidemiology Cincinnati Children's Hospital Medical Center Cincinnati, OH United States; 4 The Christ Hospital Health Network Cincinnati, OH United States; 5 Division of Asthma Cincinnati Children's Hospital Medical Center Cincinnati, OH United States; 6 Precision Pulmonary Medicine Program, Department of Medicine Indiana University School of Medicine Indianapolis, IN United States

**Keywords:** stress, immigrant health, DNA methylation, cumulative stress burden, chronic illness, Ethiopia

## Abstract

**Background:**

Immigrants experience significant health disparities, which are exacerbated by a heavy stress burden, which in turn affects the epigenetic profiles of the immune system, leading to chronic diseases. Cumulative stress burden for immigrants ranges from immigration-related stressors to unique psychosocial, environmental, and everyday challenges, all of which contribute to negative psychological and biological impacts on their health over the life-course. Although social and environmental conditions have been established as key factors driving disparities in health outcomes, the effects of stress and epigenetic change among immigrants remain poorly understood, impeding the development of novel and robust intervention approaches aimed at reducing health disparities. Epigenetic changes can act as surrogate markers for the stress effect. However, very few studies have examined epigenetic marks associated with stress among African immigrants. Ethiopians form one of the largest groups of African immigrants in the United States; yet, this is the first study of this kind among Ethiopian American immigrants.

**Objective:**

This protocol aims to quantify cumulative stress burden and determine DNA methylation (DNAm) associated with stress among Ethiopian American immigrants.

**Methods:**

Working with community partners, a community advisory board, and 3 community research coordinators, we will conduct a community-engaged research study of Ethiopian American immigrants. Data collection occurs at public events, church gatherings, and festivals. We use a multistation model composed of five stations through which participants rotate: (1) eligibility screening and consent, (2) stress-related questionnaires, (3) clinical measures, (4) health status and demographic questionnaires, and (5) saliva collection and incentive receipt. The measures used are: Stress of Immigration Survey, Boen’s stress exposure measure, 10-item Perceived Stress Scale, Patient-Reported Outcomes Measurement Information System Global Health Scale (version 1.2), 8-item Patient Health Questionnaire, and 7-item Generalized Anxiety Disorder. Latent profile analysis, chi-square tests, and logistic regression will be used. Saliva samples will be tested using genome-wide DNAm. With a subset of the sample, we will also conduct and thematically analyze qualitative interviews to understand additional experiences of stress and buffers to stress among participants.

**Results:**

This study received National Institutes of Health R21 grant funding. Data collection began in October 2024 and will continue until November 2025 (currently at 89% completion). In November 2025, we will start data cleaning and analysis of questionnaires, clinical measures, and DNAm. We plan to complete data analyses and prepare scientific and community outputs by the Spring of 2026.

**Conclusions:**

The study will provide new insights to address health disparities among growing immigrant populations in the United States, informing novel and robust intervention approaches to reduce chronic illness and associated sequelae for vulnerable populations. Findings from this study may highlight the need for policy change, such as providing more support in the form of infrastructure and social service agencies for immigrants.

**International Registered Report Identifier (IRRID):**

DERR1-10.2196/85971

## Introduction

Stress is ubiquitous for immigrants and ranges from daily stressors to unique psychosocial, environmental, and acculturative challenges contributing to adverse psychological and biological impacts on their health over the life-course [1-4]. Immigration-related stress includes acculturative stress compounded by language, financial, and isolation barriers [5] and the pressure of sending remittances to their native country [6,7]. Some immigrants have undergone challenging and traumatic journeys to the United States or, in their native country, experienced traumatic events (premigration stress) [6-9]. Additionally, an anti-immigrant sociopolitical environment in the new host country can exacerbate stress for immigrants [10-14]. Immigrants can also experience other forms of psychosocial stress and adversities from “everyday stressors” of family, work, acute stressful events, as well as social determinants that contribute to ill health (eg, poverty and community violence) [7,15-17]. In nontraditional migration areas like the Midwest, there is less infrastructure and social service agencies to support immigrants, less social acceptance of immigrants in general [18], and fewer ethnic enclaves, which often provide social support to immigrants [19], all of which can contribute to further exacerbation of stress. Taken together, immigrants may experience multiple types and sources of stress that accumulate to impact health risk and disease [20-24] and contribute to the decline of the initial immigrant health advantage [25,26]. It is well established that stress experiences and adversities have significant health consequences in a dose-response relationship such that cumulative exposure to stress, regardless of type or source, is associated with decrements and disparities in physical and mental health for vulnerable and minority groups such as immigrants [22-24,27,28]. To better understand immigrant-related stress processes, studies that assess immigrant stress burden multidimensionally.

Epigenetic changes are heritable; thus, they represent a key biological mechanism through which the effects of stress can be transmitted across generations [29,30]. This intergenerational transmission is further shaped by environmental factors that influence stress and, consequently, immune-related epigenetic changes [31,32]. Mounting evidence suggests that changes in the environment alter stress levels, which in turn affect the host epigenome profiles of the immune system, leading to health-related diseases [33]. There is growing evidence suggesting that immigration-related stress can leave measurable epigenetic signatures, particularly in DNA methylation (DNAm) patterns of stress-response genes [31,34]. For instance, psychosocial stressors in Latino immigrants have been associated with altered methylation profiles related to cardiometabolic risk [35]. Additionally, epigenetic modifications in DNAm have been recently documented with Syrian refugees experiencing war-related violence [31]. Hence, epigenetics is the missing link between migration-related experience and diseases outcome [36]. Several studies have supported epigenetics as a mechanism linking immigrant stress and cardiometabolic diseases (obesity, diabetes, hypertension, and stroke) and mental disorders (depression and anxiety disorders) [35-44]. Higher levels of cumulative family adversity, characterized by co-occurring stressors in a family context, were associated with DNAm at 7 sites, primarily in stress- and immune-related genes [45]. While stress has been suggested to induce epigenetic modifications resulting in racial differences in stress [46], studies are lacking in immigrant populations.

Within the United States, Black African immigrants experience disproportionate economic, health, and social disadvantage compared to other immigrant groups [47]. African immigrants have been referred to as “invisible immigrants” in the United States because they are rarely included in immigration policy discussions or research studies on immigrants [48,49]. Although not monolithic, Black African United States immigrants face the likelihood of compounded stress resulting from additional challenges associated with socioeconomic disadvantage, racism, and acculturation [50]. Thus, research is lacking on health care needs, priorities, and challenges experienced by this significantly growing segment of the US immigrant population, particularly in nontraditional migration areas. Currently, the second largest African immigrant population in the United States [48,51], Ethiopian American immigrants in the United States have steadily increased over the years. The United States is the most common destination for Ethiopian immigrants and a top source for remittances to Ethiopia [52]. Despite the large number of Ethiopian immigrants living in the United States, limited health research has been conducted with this population [6].

**Figure 1:**
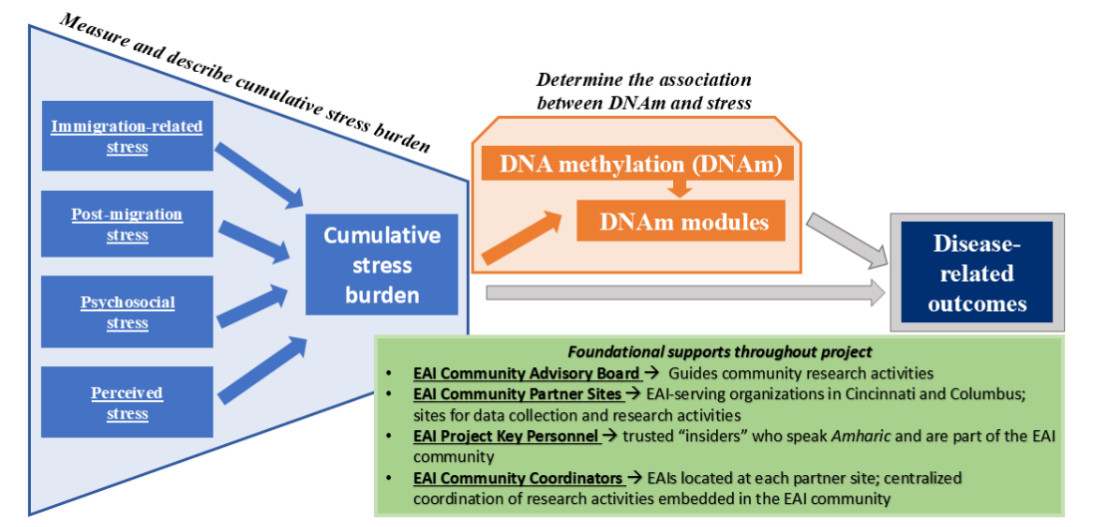
Conceptual model of the proposed research. EAI: Ethiopian American immigrant.

In collaboration with our established Ethiopian American immigrant Community Advisory Board (CAB) and community partners, our team recently conducted a community health needs assessment of Ethiopian immigrants in Cincinnati and Columbus, OH, funded by the Cincinnati Academic Health Clinical Translational Science Awards Center [53]. Understanding the health beliefs, behaviors, needs, and priorities of Ethiopian American immigrants informs and supports this study. Importantly, we established an Ethiopian American immigrant CAB that met monthly during the needs assessment. Building on our previous work in immigrant health and this recent health needs assessment of local Ethiopian American immigrants, our goal is to better understand how psychological, social, environmental, and acculturative stressors affect the risk for chronic diseases through epigenomic mechanisms. Figure 1 outlines the conceptual model and purpose of this study, which is to (1) measure and describe cumulative stress burden and (2) determine the association between DNAm and stress among Ethiopian American immigrants. In secondary analyses, we will also examine the association of stress and specific chronic diseases—obesity, diabetes, hypertension, depression, and anxiety. This study directly addresses gaps in the literature investigating the multiple pathways through which cumulative stress becomes embodied and “gets under the skin” of Ethiopian American immigrants. This study will set the stage for action to address health disparities, including future interventions that are contextually grounded in the everyday realities of Ethiopian American immigrants’ lives.

## Methods

### Overview and Study Design

In this biopsychosocial cross-sectional and qualitative study, we use a community-engaged research approach [54] working with Ethiopian community partners at each step of the research process. Data collection occurs in the community at public events (eg, health fairs, festivals, and meetings) using a multistation model comprised of five stations through which participants rotate: (1) eligibility screening and consent, (2) stress-related questionnaires to assess cumulative stress burden, (3) clinical measures (hemoglobin A_1c_ [HbA_1c_], height, weight, and blood pressure [BP]), (4) health status and demographic questionnaires, and (5) saliva collection and incentive receipt. See supplementary files for Observational Study Protocol and Qualitative Study Protocol Checklists [55].

### Study Setting

To capture a range of Ethiopian American immigrant experiences, data collection will occur with Ethiopian American immigrant community samples in 2 geographic areas in the Midwest: Greater Columbus and Greater Cincinnati, Ohio. These Midwest cities have not traditionally been destinations for immigrants, and culturally and linguistically appropriate social services and social networks are less established.

### Community Partners

To ensure that our study is relevant and feasible for Ethiopian American immigrants and for a wider translation to the community, we are partnering with 2 Ethiopian American immigrant–serving community sites- an Ethiopian social service agency in Columbus and an Ethiopian Orthodox church in Cincinnati. We are working with an established CAB of Ethiopian leaders and 3 Ethiopian community research coordinators (BA, BG, and HE) hired for this study.

A CAB already established for a previous study will continue to meet for this study. The CAB has 6 members with representation from each of the 2 community partner sites. The CAB will help translate measures into Amharic and ensure the project is culturally and contextually relevant, sensitive, and feasible for Ethiopian American immigrants. The CAB will meet with the research team at least quarterly to provide input on study progression to ensure the project is relevant and meaningfully engaging to the Ethiopian American immigrant communities, review study materials, and interpret and disseminate findings. CAB members will be compensated with US $50 for attendance at each CAB meeting.

The Ethiopian community research coordinators have relevant knowledge of health and community services and have received formal research training from the principal investigators to coordinate data collection. Community research coordinators have been trained in the appropriate collection of biometric screening tests for hypertension, diabetes, and obesity. The training sessions integrated and balanced the cultural and contextual expertise of the community research coordinators and the research expertise of the academic members. The community research coordinators hired for this study completed CIRTification, which is a training program in human research protections that is tailored to the unique roles of community research partners [56].

### Study Sample

Over 12 months, we will recruit and enroll 150 Ethiopian American immigrant (approximately 75 per site) participants from 2 Ethiopian American immigrant–serving community partner sites in Columbus and Cincinnati, Ohio. Recruitment, consent, and conduct of the research will occur in collaboration with each site at their sites and associated public events (eg, health fairs and festivals). At each public data collection event, we will use maximal variation sampling [57] to identify potential participants of diverse ages, genders, and varied immigration length and status. After a potential participant is identified, we will ask screening questions to determine eligibility. Eligible participants must be (1) older than >18 years, (2) Ethiopian American immigrants born in Ethiopia, and (3) able to speak in Amharic or English.

### Data Collection

#### Overview

At each public data collection event, eligible participants, in their preferred language (ie, English or Amharic) will receive a full verbal explanation of the study (ie, information about the study, eligibility criteria, compensation, benefits and risks to study participation, steps to ensure data confidentiality, and right to withdraw from study at any time) and written informed consent will be obtained prior to participation. Participants can ask questions about the study to a study team member, and if they agree to participate, sign the informed consent document (Station 1). Proceeding through the remaining stations, participants will complete verbally administered questionnaires (stress measures, health status, and demographics), provide biological samples (ie, finger prick point of care HbA_1c_ and saliva for DNA), and complete screening tests for obesity, hypertension, diabetes, depression, and anxiety (Table 1).

**Table 1:** Study measures.

Categories			Variables		Measures
**Cumulative stress burden**					
	To capture both the unique and joint contributions of stress burden for EAIs^a^, we will measure cumulative stress burden in a robust, multidimensional way, which includes validated stress scales assessing immigration-related stress post migration stress, and psychosocial perceived stress (recent and chronic stress).	Immigration-related stress Post migration and psychosocial stress Perceived stress		21-item SOIS^b^ 36-item Boen’s stress exposure measure PSS-10^c^	
**Health conditions and health status**					
	Estimates of perceived and actual health status will be obtained using self-reported measures of health status and screening tests for obesity, diabetes, hypertension (high BP^d^), depression, and anxiety.	Perceived health status Overweight, obesity Hypertension Diabetes Depression Anxiety		10-item PROMIS^e^ Global Health Scale (v1.2) Weight, height, and BMI Systolic and diastolic BPs Glycosylated HbA1c^f^ PHQ-8^g^ GAD-7^h^	
**DNA methylation**					
	We will develop the WGCNA^i^ module associated with stress burden variables and identify methylation modules and functional pathways.	DNA		Saliva samples or oral-rinse specimens will be obtained by a trained study team member	
**Demographics**					
	10 items regarding family and background	—		—	

^a^EAI: Ethiopian American immigrant.

^d^SOIS: 21-item Stress of Immigration Survey.

^e^PSS-10: 10-item Perceived Stress Scale.

^b^BP: blood pressure.

^f^PROMIS: Patient-Reported Outcomes Measurement Information System.

^g^HbA_1c_: hemoglobin A_1c_.

^h^PHQ-8: 8-item Patient Health Questionnaire.

^i^GAD-7: 7-item Generalized Anxiety Disorder.

^c^WGCNA: weighted gene co-expression network analysis.

Data are anonymous, and each participant will receive a unique ID number, which is used on all measures. All study data and tracking will be entered into the REDCap (Research Electronic Data Capture; Vanderbilt University) database, a secure research electronic data management system that provides an interface for validated data entry, audit trails for tracking data manipulation, and export procedures. REDCap is compliant with the HIPAA (Health Insurance Portability and Accountability Act) and satisfies all local, state, and federal regulations for the capture and storage of private health information for research purposes.

Participants who express any distress during verbal administration of questionnaires, those who indicate high levels of stress, and those with abnormal or elevated clinical measures will be referred, and the on-call physician will determine the need for emergency room evaluation. Participants with an abnormal HbA_1c_ result (≥5.7), elevated BMI (≥25), and/or elevated BP (≥120/80 mm Hg) will be counseled on healthy lifestyle modifications and the need for medical evaluation with a primary care provider. Participants will then be provided with contact information for local primary care clinics. If a participant has a screening test that requires urgent or emergent evaluation (HbA_1c_ >9, random blood glucose of ≥ 400, and/or BP ≥180/120 mm Hg), the community coordinator will contact the on-call physician for consultation. The on-call physician will speak with the participant and determine the need for emergency room evaluation. Participants who complete the data collection event will receive a US $75 gift card.

#### Cumulative Stress Burden

To capture both the unique and joint contributions of stress burden for Ethiopian American immigrants, we will measure cumulative stress burden in a robust, multidimensional way [58] which includes validated stress scales assessing immigration-related stress, post-migration stress, and psychosocial perceived stress (recent and chronic stress) in combination with a saliva specimen (biological measure of stress) and qualitative semistructured interviews to capture additional missed experiences of stress and buffers to stress. The scales and interviews will be verbally administered by the trained community research coordinators. We will work with the CAB and a professional translator to translate scales and the interview guide into Amharic. We will work with the CAB to ensure that all measures are culturally appropriate for Ethiopian American immigrant.

Immigration-related stress will be measured using the 21-item Stress of Immigration Survey (SOIS), which assesses 5 domains of stress: language, immigrant status, work issues, yearning for family and home country, and cultural dissonance among immigrants living in the United States [59]. The SOIS has robust psychometric properties with immigrant samples. Post migration, psychosocial, and perceived stress will be assessed using Boen’s stress exposure measure [20] which considers stress and adversity in 6 domains of social life: lifetime traumas; recent stressful life events (last 5 y); financial strain; ongoing chronic strains (eg, health, work, relationship, and housing); everyday discrimination; and major lifetime discrimination. The individual measures have been previously used to assess the association between psychosocial stress and a variety of mental and physical health outcomes (eg, depressive symptoms, chronic illnesses, and physical limitations) [21]. Following the same procedure used by Boen and Hummer [20] and Cuevas et al [27], we will create 2 measures of cumulative exposure across these stress domains: (1) high risk stress burden (numbers of stressors for which the respondent is in the highest quartile and (2) composite total stress exposure across domains of stress (no cutoffs). The 10-item Perceived Stress Scale (PSS-10) is a 10-item scale that is used to assess the degree of perceived stress a person experiences [60]. The items relate to how unpredictable, uncontrollable, and overloaded respondents find their lives in general and do not focus on a specific event. The PSS-10 has been used in multiple languages and has well-established psychometric properties, including in Ethiopian university students [61-64]. A biological measure of stress will be collected with saliva samples or oral-rinse specimens obtained by trained study team members. Nonfasting saliva will be collected from each participant following oral rinsing with tap water. Participants will expectorate into an Oragene-RNA RE-100 Self-Collection Kit (DNA Genotek), and samples will be stored at room temperature for further analysis [65]. Buccal cells for DNA extraction will be collected using DNA Genotek saliva collection kits [66]. Saliva has been used as a noninvasive and stress-free diagnostic alternative to blood to examine associations of stress and resilience factors with DNAm by our groups and others [37,67,68].

#### Health Status and Health Conditions

Estimates of perceived and actual health status will be obtained using self-reported measures of health status and screening tests for obesity, diabetes, hypertension (high BP), depression, and anxiety. Perceived health status will be assessed with the Patient-Reported Outcomes Measurement Information System (PROMIS) Global v1.2 scale [69-71], a self-reported measure of global, physical, mental, and social health. Participants’ BMI will be measured and used to categorize participants’ weight status. Screening for hypertension will be conducted using measurements of systolic and diastolic BP. BP measurements will be used to categorize participants into categories (normal, elevated, stage 1 hypertension, and stage 2 hypertension). Screening for diabetes will be conducted using a point-of-care capillary blood glucose test (HbA_1c_), which will be used to categorize participants (normal blood sugar, prediabetes, or diabetes). Depression and anxiety symptoms will be measured using the 8-item Patient Health Questionnaire (PHQ-8) and the 7-item Generalized Anxiety Disorder (GAD-7) assessments, both widely used and well-validated in different settings and population groups [72-75]. PHQ-8 and GAD-7 have been validated in Ethiopia using different cutoff points (5 or above on the PHQ-8; 10 or above on the GAD-7) [76-78].

#### Qualitative Interviews

To explore the nuances of Ethiopian American immigrant stress burden, trained members of the team will conduct qualitative semistructured interviews [79] with a subset of Aim 1 Ethiopian American immigrants who vary in levels of reported stress on the above measures. These interviews will be conducted to ensure we understand Ethiopian American immigrant experiences with stress that may be missed from standardized measures and to explore potential buffers to the adverse effects of stress (Multimedia Appendix 1 for interview guide reviewed and pilot tested with members of the CAB). We will contact participants by phone, text, or WhatsApp (Meta; depending on preference indicated during the public data collection events) and schedule interested individuals for a ~60-minute web-conference (eg, Zoom and Microsoft Teams) interview. We will use maximal variation sampling [57] to identify potential interviewees of diverse ages, genders, and varied immigration length and status. Data collection will continue until saturation, or informational redundancy [80-82] of major categories and themes is achieved. Specifically, we will operationalize saturation, for the purpose of this study, as the point at which no new substantive information is gained from 3 consecutive interviews. We will conduct a minimum of 10 interviews in each geographic area (Cincinnati and Columbus) and expect that we will reach data saturation within 20-25 total interviews based on our prior experience in similar qualitative projects with immigrant populations.

### Data Analysis

We will use latent profile analysis (LPA) [83,84], combining all the stress subscales to identify, within the study sample of Ethiopian American immigrants, subgroups having similar within-group, but different between-group profiles of cumulative stress. When combined with epigenetic profiling and network-based methylation module analysis, a multidimensional understanding of stress is achieved. Instead of using a single-variable analysis, this method yields a systems-level perspective of how stress may shape immune-related gene activity and how it contributes to immigrants’ vulnerability to disease. This type of analysis examines whether DNAm modules mediate the relationship between stress and chronic health conditions, including obesity, diabetes, hypertension, depression, and anxiety.

Prior to LPA, descriptives for the stress subscales will be generated, and normality, skewness, kurtosis, or outliers will be examined. Z-standardized mean subscale scores of all measures will be generated and used in LPA. As all measures are continuous variables, the maximum likelihood approach or maximum likelihood with robust SEs will be used for LPA. The number of profiles will rely on statistical fit, as well as theoretical and content-related considerations. Models with different numbers of latent profiles will be fitted using a stepwise approach, starting with 2 profiles and successively adding profiles. In each step, we will examine the fit information criteria (Bayesian information criterion, [85] sample-size adjusted Bayesian information criterion, [86] and Akaike information criterion [87,88]). To compare models with one fewer or more profiles, we will apply the bootstrapped Likelihood Ratio Test [89,90] and the adjusted Lo-Mendell-Rubin test [91]. A profile that includes <1% of the total sample size or fewer than 25 cases will not be considered to avoid lower power, precision, and less parsimony. Profiles will be inspected for theoretical and content-related considerations and assigned labels. An alternative approach for LPA will be to use one measure of cumulative stress (eg, Stress of Immigration Survey). Missing data is assumed missing at random. Missing data will be examined for patterns and handled using either the full information maximum likelihood estimation method or multiple imputation. Based on past research and rules of thumb, the simulation study of Nylund et al [92] concluded that a smaller sample size may be adequate with simpler models and well-separated profiles.

### Secondary Analyses A

Association between the composite stress burden and chronic physical and mental health conditions. In a secondary analysis using chi-square tests and logistic regression with adjustment for covariates, the identified latent stress burden profiles will be tested for associations with physical and mental health conditions for which we screened (obesity, diabetes, hypertension, depression, and anxiety). This also serves as validation of the profiles. We will also examine how the identified profiles are related to demographics and other covariates, and whether and how latent stress burden profiles differ by cities.

#### Genome-wide DNAm

We will use a genome-wide approach (Illumina EPIC arrays) to identify Cytosine-phosphate-Guanine (CpG) sites associated with stress and chronic illnesses (hypertension, obesity, cardiovascular disease, and diabetes). One-microgram DNA from saliva samples will be treated with bisulfite and submitted to the DNA Sequencing and Genotyping Core for DNAm quantification. The genome-wide DNAm analysis will be performed using the Infinium Human Methylation 850 BeadChip “860K array” (Illumina), [93] which covers 99% RefSeq genes, 333,265 CpG sites located in enhancer regions identified by the ENCODE (Encyclopedia of DNA Elements), 5’ untranslated region (5’UTR), exon, gene, and 3’ untranslated region (3’UTR) regions [94]. The 850K array has recently become an attractive choice for DNAm studies due to its effective cost and high coverage of promoter regions [95]. Samples will be randomly assigned to avoid batch effects due to chips and plates. The MethylationEPIC BeadChip images will be captured using the Illumina iScan. Samples with high-quality bisulfite conversion efficiency (intensity signal >4000) that also pass all GenomeStudio quality control steps based on built-in control probes for staining, hybridization, extension, and specificity will be filtered. Individual probes will be filtered away based on mean detection *P*>.01. The raw DNAm data will be exported from GenomeStudio and analyzed using Bioconductor and the lumi package.

#### Single CpG Site Analysis

Briefly, signal intensities of samples that pass quality control will be background-adjusted using out-of-band probes (noob) and normalized using subset-quantile within array normalization (“swan”). Beta values calculated as signal_methylation_/(signal_methylation_+signal_unmethylation_) will be used as the measure for DNAm levels, while m values will be used in the analysis. Since DNAm is collected from saliva, and it may be affected by cell composition [96] and cell dilution, we will correct for cell composition using Houseman reference-based deconvolution algorithms (R package “minfi”). We will subsequently control for possible batch effects, as well as hidden structure in DNAm data, using surrogate variable analysis. To test association of DNAm with stress, we will use linear regression models (R limma package), [97] where DNAm level of a CpG locus will be the dependent variable, stress factors in aim 1 are the independent variables and the following covariates will be adjusted: significant surrogate variables from surrogate variable analysis, estimated proportions of saliva cell types, immigration duration, age, and sex, as well as a random effect for recruitment site. Differentially methylated CpG sites with methylation differences of at least 5% and *P*<.0001 will be identified.

#### Construction of Methylation Networks and Identification of Methylation Modules

Because exposure to cumulative stress most likely affects methylation of a variety of genes, single CpG site analysis may fail to fully capture the epigenetic pathways impacted by stress. We plan to use weighted gene coexpression network analysis (WGCNA) to identify methylation modules (ie, groups of genes with highly correlated methylation values and that may represent biological pathways) and then relate these modules to stress profiles. We apply the coexpression approach because differentially expressed genes may fail to fully capture the molecular pathways, as genes do not function in isolation [98]. Briefly, WGCNA takes the methylation correlation matrix and transforms it into a weighted undirected network by raising the absolute value of each pair-wise correlation to a power β. The higher the value of β, the more weight is given to high correlations at the expense of low correlations [99]. This weighting scheme preserves the continuous nature of the methylation data and produces highly robust results. Modules are then identified using average linkage hierarchical clustering. In addition, the use of network approaches reduces the burden of multiple testing, as we will test the association of a few hundred modules, representing biological pathways, with the stressors of interest, rather than test hundreds of thousands of methylation sites. TM and LD have conducted WGCNA-related experiments in the past [98,100].

#### Cumulative Stress - DNAm Modules Association Analysis

We will calculate for each module the eigengene E that is defined as the first principal component of the methylation values of a given module. The epigenome can be considered as representative of the methylation profiles in a module. The association between the identified modules and cumulative stress can then be evaluated using linear models. Analyses will be adjusted for principal components of methylation variation (calculated from the epigenome-wide methylation data), estimated proportions of saliva cell types, other covariates (eg, immigration duration, age, and sex), and random recruitment site effect. We will also examine what genes and/or CpG sites have a greater contribution to the stressor-module association by looking into their weights on the module eigengene.

### Secondary Analyses B

Determine the mediation effects of DNAm modules between stress profiles and specific outcome Studies have shown an association between stress and health outcomes. However, none of these studies has examined whether health outcomes in immigrants are potentially mediated by DNAm modules. A mediation analysis will be performed to assess whether or not the observed association between stress and health outcome is mediated through DNAm modules. Mediating variables are selected on sound theories that provide evidence for pathways of relationship [101-103].

#### Testing for DNAm Modules Mediation Effects

Our mediation analysis aims to explore the relationship between the independent variable (stress, X), the mediator (DNAm, M), and the dependent variable (disease-related outcome, Y) [102-104]. The test for mediation will be accomplished with a 3-step model. We will include covariates (age, sex, and immigration duration) in all of the models. However, for simplicity, we will omit them in the following equations: Step 1: logit(Pr(Y=1, x)) = ɑ_1_ + C’X, testing for path C’ (total effect) when X predicting Y; Step 2: M = ɑ_2_ + AX + ε_2_, testing for path A when X predicting M; and Step 3: logit(Pr(Y=1|x, m))= ɑ_3_ + BM + CX, multiple regression analysis with X and M predicting Y. The direct effect (path C) is the effect of stress on health outcome, adjusting for the mediator (methylation modules). The mediation effect is the indirect effect (path A*B) [105]. We will estimate the average causal mediation effects [106].

#### Power Analysis

Power analysis is based on the association between DNAm module eigengenes and cumulative stress burden. We expect to analyze about 100 normalized module eigengenes from 100 methylation modules using genome-wide methylation data. With a sample size of 150, for a multiple linear regression model which already includes ~6 covariates with a squared multiple correlation ρ² of 0.1, a sample size of 150 will have 80% power to detect at a significance level of 3.125×10^–5^ (=0.05/100 methylation modules × 16 stress subscales) an increase in ρ² of 0.136 due to including 1 additional stress subscale.

### Qualitative Analysis

We will use an inductive process of thematic analysis [107,108] to code the interview data to the point of saturation (described above). Guided by an expert in qualitative research (LV), we will use Dedoose, a qualitative software for data coding and management [109]. At least two study team members with prior experience in qualitative research and study-specific training will double-code each interview, modifying the coding structure as needed. Using established standards for qualitative research [110,111], the coding will be compared, and disagreements resolved through discussion. As coding progresses, individual concepts will be grouped into categories and eventually themes. Once we have preliminary themes, we will use a “reflexive participant collaboration” approach [112] and conduct validation (peer review) interviews [113] with members of the CAB and additional participants.

### Ethical Considerations

The institutional review board at Cincinnati Children’s Hospital Medical Center approved this study (#2024-0176). Informed consent in the participant’s preferred language (English or Amharic) was obtained for all participants. The study data are deidentified and entered directly into a password-protected REDCap database. Only study staff have access to this database. Participants are compensated via a reloadable debit card. They receive US $75 for initial data collection and US $40 if selected for a qualitative interview. CAB members receive US $50 for their participation in meetings.

## Results

**Figure 2:**
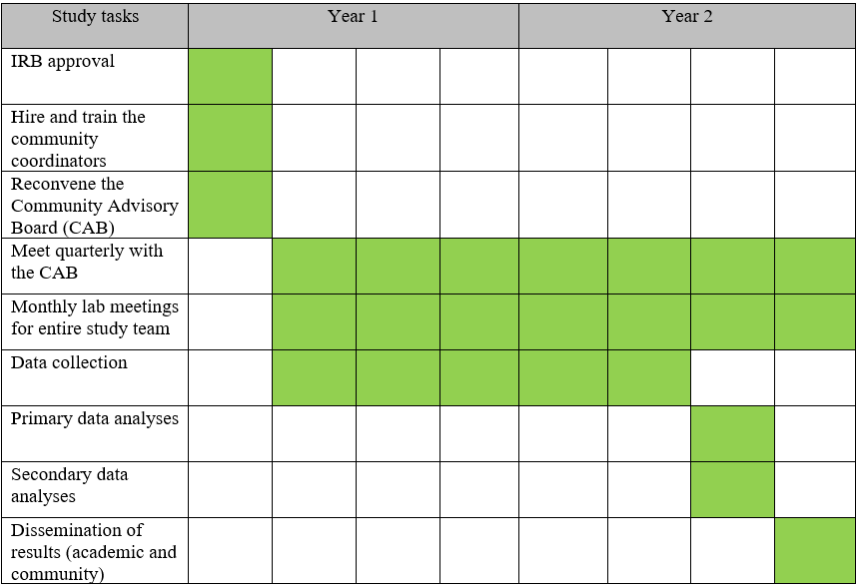
Study timeline.

The study will span 2 years (Figure 2). After receipt of funding, the first 3 months were devoted to institutional board review approval, hiring and training community research coordinators, reconvening the CAB from a previous project, ordering data collection supplies (eg, clinical tests and tools and saliva collection tools), and initial planning conversations with community partners regarding recruitment, challenges, and data collection strategies. October 2024 to November 2025 will be focused on data collection (currently at 89% completion). In November 2025, we will start data cleaning and analysis of questionnaires, clinical measures, and DNAm. We have begun qualitative interviews with a subset of the participants to understand additional experiences of stress and buffers to stress among participants. Although data analysis has not started, preliminary insights from the study include participants’ discomfort and unwillingness to use the word stress in stress-related questionnaires and interviews, and instead emphasizing the words “worry” and “concerns,” and perceived stigma of naming and/or identifying with mental health disorders such as depression and anxiety on the PHQ-8 and GAD-7. The importance of religion, prayer, turning to God, and reliance on priests has consistently been emphasized by participants during data collection and interviews. Social connections within and across Ethiopians living in Cincinnati and Columbus versus non-Ethiopians seem to be prioritized based on participants’ responses to the questionnaires and in the interviews thus far. We plan to complete all data analyses and prepare scientific and community outputs by Spring 2026.

## Discussion

### Anticipated Findings

This study protocol investigates how cumulative stress burden affects chronic disease risk among Ethiopian American immigrants. It examines how cumulative stress uses both psychosocial and biological pathways. To this aim, we used a combination of validated stress measures, clinical health indicators, and genome-wide DNAm analysis. The framework for this study acknowledges that the stress experienced by immigrants is complex and can include premigration trauma, postmigration challenges, and sociopolitical adversity. Compounding the mental health risks of stress among immigrants is a significant disparity in adequate treatment for depression, mental health concerns, and substance abuse [114-116]. Barriers to access and quality mental health care for immigrants are multifactorial and include complex drivers across societal and individual levels [114,117-120]. Barriers at the societal and structural level include lack of health insurance, low socioeconomic status, limited English proficiency, low educational attainment, documentation status, logistics, scheduling, and culturally and linguistically congruent health care providers [114,119,121-125]. At the individual level, cultural mistrust of the mental health system, perceived discrimination from health care providers, focus on physical versus psychiatric symptoms, and cultural stigma can lead immigrants to attempt to manage stress and mental health issues on their own [11,114,119,126-131]. Because of the increased risk of stress burden, immigrants are in dire need of secondary prevention via mental health support that is accessible, asset-based, and culturally relevant [50]. Without such support, stress burden and barriers to mental health care experienced by many immigrants increase the risk of suicide, addiction, chronic disease, and early death [124,128,132-141]. It is important to note that the study’s community-engaged approach fosters cultural sensitivity, builds trust within the Ethiopian American immigrant community, and strengthens recruitment and retention efforts among a population that is typically difficult to study through conventional research strategies [53,142].

Our initial qualitative findings indicate that the way stress is expressed and understood, such as the culturally preferred terms like “worry” or “concern” instead of stress, along with the influence of religious beliefs and intraethnic social ties, are essential for capturing how Ethiopian American immigrants experience and interpret stress. This knowledge will assist in the interpretation of quantitative results and will inform future interventions. US Black African immigrants have been underrepresented in research about health. The results of the study will make an important contribution to the literature on immigrant health disparities by focusing on Black African immigrants from Ethiopia. The use of epigenetic data gives us the opportunity to study how stress may affect us biologically, and whether it may be transmitted across generations. Results of this study will inform the development of culturally relevant interventions that can positively impact the health and well-being of US immigrants.

### Limitations

While the study offers substantial strengths, several limitations should be acknowledged. The participant pool is restricted to Ethiopian American immigrants living in Columbus and Cincinnati, Ohio—areas considered nontraditional migration destinations with distinct social environments. As a result, the findings may not be broadly applicable to Ethiopian American immigrants in other regions with differing sociopolitical contexts, community structures, or support systems. Additionally, the study design limits the ability to draw causal conclusions. Although mediation analyses are planned, longitudinal data will be essential to establish temporal relationships between stress exposure, DNAm changes, and health outcomes.

Many of the study’s measures rely on self-reported data, which may be affected by language barriers and cultural stigma, especially regarding mental health. While saliva collection is a convenient and noninvasive method suitable for community-based research, it may not fully reflect methylation patterns in specific tissues involved in chronic diseases. Furthermore, although the study is adequately powered to examine stress profiles and their associations with health indicators, the sample size may be relatively small for genome-wide DNAm analyses—particularly when detecting small effect sizes or conducting subgroup analyses.

### Comparison With Prior Work

This study builds on our previous work in immigrant health and a recent health needs assessment of local Ethiopian American immigrants [53].

### Conclusions

This study is innovative in its exploration of existing health disparities among immigrant populations in the United States. Results may offer valuable insights that support the development of new and effective intervention strategies to reduce chronic illness in these vulnerable communities. Specifically, findings from this study may highlight the need for policy change, such as providing more support in the form of infrastructure and social service agencies to support immigrants.

## Appendix

Multimedia Appendix 1 Peer review report from MESH - Biobehavioral Mechanisms of Emotion, Stress and Health Study Section, Biobehavioral and Behavioral Processes Integrated Review Group (National Institutes of Health, USA).

## Abbreviations

BP: blood pressure
CAB: Community Advisory Board
CpG: cytosine-phosphate-guanine
DNAm: DNA methylation
ENCODE: Encyclopedia of DNA Elements
GAD-7: 7-item Generalized Anxiety Disorder
HbA1c: hemoglobin A1c
HIPAA: Health Insurance Portability and Accountability Act
LPA: latent profile analysis
PHQ-8: 8-item Patient Health Questionnaire
PROMIS: Patient-Reported Outcomes Measurement Information System
PSS-10: 10-item Perceived Stress Scale
REDCap: Research Electronic Data Capture
SOIS: Stress of Immigration Survey
WGCNA: weighted gene co-expression network analysis

## Data Availability

The datasets generated or analyzed during this study will be available from the principal investigators (TM and LV) on reasonable request once the study is completed.
