# Correction: Cumulative Stress Burden and Association With DNA Methylation in Ethiopian American Immigrants: Protocol for a Community-Engaged, Biopsychosocial Study

**DOI:** 10.2196/95656

**Published:** 2026-03-31

**Authors:** Lara Stone, Lili Ding, Meron Hirpa Kassa, Beakal Amsalu, Bruktawit Getnet, Hermella Eshete, Tesfaye B Mersha, Lisa M Vaughn

**Affiliations:** 1 Division of Emergency Medicine Cincinnati Children's Hospital Medical Center Cincinnati, OH United States; 2 Department of Pediatrics College of Medicine University of Cincinnati Cincinnati, OH United States; 3 Division of Biostatistics/Epidemiology Cincinnati Children's Hospital Medical Center Cincinnati, OH United States; 4 The Christ Hospital Health Network Cincinnati, OH United States; 5 Division of Asthma Cincinnati Children's Hospital Medical Center Cincinnati, OH United States; 6 Precision Pulmonary Medicine Program Department of Medicine Indiana University School of Medicine Indianapolis, IN United States

In “Cumulative Stress Burden and Association With DNA Methylation in Ethiopian American Immigrants: Protocol for a Community-Engaged, Biopsychosocial Study” [1] the authors noted two errors.

The seventh author (TBM) has added the middle initial “B” to their name.

In addition, the following affiliation has been added as affiliation 6 and linked to TBM:

Precision Pulmonary Medicine Program, Department of Medicine, Indiana University School of Medicine, Indianapolis, IN

The correction will appear in the online version of the paper on the JMIR Publications website, together with the publication of this correction notice. Because this was made after submission to PubMed, PubMed Central, and other full-text repositories, the corrected article has also been resubmitted to those repositories.
