# Supplementary material for: Cumulative Stress Burden and Association With DNA Methylation in Ethiopian American Immigrants: Protocol for a Community-Engaged, Biopsychosocial Study
Source: JMIR Res Protoc. 2026 Feb 10;15:e85971. doi: 10.2196/85971 (PMC12933171; doi:10.2196/85971)
Supplement: Multimedia Appendix 1 [file resprot_v15i1e85971_app1.pdf]

SUMMARY STATEMENT
(Privileged Communication)

PROGRAM CONTACT:
Gabriel Lai
(301) 402-1366
gabriel.lai@nih.gov

Release Date: **07/24/2023**
Revised Date:

Application Number: **1R21MD019192-01**

Principal Investigators (Listed Alphabetically):

MERSHA, TESFAYE B. (Contact)
VAUGHN, LISA MICHELLE

Applicant Organization: CINCINNATI CHILDRENS HOSP MED CTR

Review Group: **MESH**
Biobehavioral Mechanisms of Emotion, Stress and Health Study Section

Meeting Date: **06/28/2023**
Council: **OCT 2023**
Requested Start: **09/01/2023**
Opportunity Number: **PAR-20-150**
PCC: **IBB01GL**
Dual IC(s): **LM**

Project Title: **Association of DNA Methylation and Stress in Immigrants**

SRG Action: **Impact Score:43 Percentile:36 +**
Next Steps: **Visit https://grants.nih.gov/grants/next_steps.htm**
Human Subjects: 30-Human subjects involved - Certified, no SRG concerns
Animal Subjects: 10-No live vertebrate animals involved for competing appl.
Gender: 1A-Both genders, scientifically acceptable
Minority: 2A-Only minorities, scientifically acceptable
Age: 3A-No children included, scientifically acceptable

Project Year	Direct Costs Requested	Estimated Total Cost
1	125,000	200,625
2	150,000	240,750
TOTAL	275,000	441,375

ADMINISTRATIVE BUDGET NOTE: The budget shown is the requested budget and has not been adjusted to reflect any recommendations made by reviewers. If an award is planned, the costs will be calculated by Institute grants management staff based on the recommendations outlined below in the COMMITTEE BUDGET RECOMMENDATIONS section.
BUDGET MODIFICATIONS

1R21MD019192-01 Mersha, Tesfaye

COMMITTEE BUDGET RECOMMENDATIONS

RESUME AND SUMMARY OF DISCUSSION: This application seeks to quantify stress burden and determine DNA methylation (DNAm) patterns associated with stress and physical health outcomes among Ethiopian American immigrants (EAIs). If successful, results from this study could clarify how changes in psychological, social, environmental, and acculturative stressors affect the risk for chronic diseases through epigenomic mechanisms. The significant disease burden and potential to inform culturally relevant interventions made this project significant; however, there was no multi-level conceptual model that illustrated the various sources of stress and the proposed epigenomic mechanisms reducing the significance. The investigative team was strong with expertise in social epigenetics, psychosocial science, social epidemiology, environmental exposure and geocoding, biostatistics, and community engaged work; however, there was a lack of stress expertise which seems key to this project. The use of weighted co-methylation network analysis and the collection of epigenetic markers from this EAI population that would enhance the genetic diversity of the field broadly were both innovative. Strengths of the approach included preliminary data that showed feasibility, data dimensionality reduction strategies, objective and subjective measures of stress and burden, existing relationships with EAI community partners, and the collection of epigenetic markers from an immigrant population from a single African country of origin. Weaknesses in the approach were highlighted during discussion, including some concerns of feasibility including a lack of details around saliva collection for DNAm sampling, important stress-related variables and conditions were not included in the modeling and there was no alternative strategy if the latent profile analysis failed, the limited focus on physical outcomes, and the validity of cultural appropriateness for the assessments was unclear. Overall, the strengths of the significance, innovation, approach were balanced with weaknesses in the significance, investigator, and approach, resulting in a moderate impact application.

DESCRIPTION (provided by applicant): Immigrants experience significant health disparities which are exacerbated by a heavy stress burden which in turn affects the epigenetic profiles of the immune system leading to chronic diseases. Stress burden for immigrants ranges from everyday stressors to unique psychosocial, environmental, and acculturative challenges, all of which contribute to negative psychological and biological impacts on their health over the life-course. Although social and environmental conditions have been established as key factors driving disparities in health outcomes, the effects of stress and epigenetic change among immigrants remains poorly understood, impeding the development of novel and robust intervention approaches aimed at reducing health disparities. Epigenetic changes can act as surrogate markers for stress effect. However, very few studies have examined epigenetic marks associated with stress among African immigrants. Ethiopians form one of the largest groups of African immigrants in the US, yet this is the first study of this kind among Ethiopian American immigrants (EAIs). The objective of this proposal is to quantify stress burden and determine DNA methylation associated with stress among EAIs. We hypothesize that the DNA methylation profile in EAIs is associated with stress. To address cultural differences and experiences and for a wider translation to the community, our interdisciplinary research team will work with relevant scientific partners connected to and/or part of EAI communities in Cincinnati and Columbus OH to achieve two Specific Aims: Aim 1) Measure stress burden and identify and define stress burden profiles among EAIs. Aim 2) Determine the association between psychosocial perceived stressors/profiles and DNA methylation. Our proposed study, involving a comprehensive set of psychosocial stressors, and genome- wide DNA methylation among an immigrant population, is the first study among Ethiopian American immigrants. The study will provide new insights to address health disparities among growing immigrant populations in the US informing novel and robust intervention approaches to reduce chronic illness and associated sequelae for vulnerable populations. Our long-term goal is to better understand

how changes in psychological, social, environmental, and acculturative stressors affect the risk for chronic diseases through epigenomic mechanisms.

PUBLIC HEALTH RELEVANCE: Immigrants experience significant health disparities which are exacerbated by a heavy stress burden which in turn affects the epigenetic profiles of the immune system leading to chronic diseases. Ethiopians form one of the largest groups of African immigrants in the US, yet this is the first study of this kind among Ethiopian American immigrants. This study will quantify stress burden and determine DNA methylation associated with stress among Ethiopian American immigrants providing new insight to address health disparities among the steadily increasing US immigrant populations.

CRITIQUE 1

Significance: 2
Investigator(s): 2
Innovation: 2
Approach: 3
Environment: 1

Overall Impact: The investigators propose to address a significant problem by implementing the proposed study. Immigrants experience significant health disparities which are exacerbated by a heavy stress burden which in turn affects the epigenetic profiles of the immune system leading to chronic diseases. One of the largest and fastest-growing groups of African immigrants in the US is Ethiopian American Immigrants. If funded, this will be the first study of this kind among Ethiopian American immigrants. This study will quantify stress burden and determine DNA methylation associated with stress among Ethiopian American immigrants providing new insight to address health disparities among the steadily increasing US immigrant populations. The team hypothesizes that the DNA methylation profile in Ethiopian American Immigrants EAIs is associated with stress. To address cultural differences and experiences and for a wider translation to the community, the interdisciplinary research team will work with relevant scientific partners connected to and/or part of EAI communities in Cincinnati and Columbus OH to achieve two Specific Aims: Measure stress burden and identify and define stress burden profiles among EAIs. This proposed study has the potential to have a significant public health impact. The investigators hope the findings of this study will identify potential targets for interventions to address stress-related chronic conditions. The team aims to elucidate how psychological, social, environmental, and acculturative stressors affect the risk for chronic diseases through epigenomic mechanisms.

1. Significance:

Strengths

- The investigators propose to address a significant problem by implementing the proposed study. Immigrants experience significant health disparities which are exacerbated by a heavy stress burden which in turn affects the epigenetic profiles of the immune system leading to chronic diseases.
- Culturally-relevant interventions are needed to reduce stress-related chronic illnesses among immigrant populations, including Ethiopian American Immigrants (EAIs).

- This proposed project has the potential to contribute to the improvement of minority health and the reduction of health disparities.
- The focus is one health disparity population that is clearly identified and justified (EAIs), and the study has implications for other immigrant populations.

Weaknesses

- No major weaknesses.

2. Investigator(s):

Strengths

- The interdisciplinary team with researchers from psychosocial science, social epidemiology, social epigenetics, environmental exposure and geocoding, biostatistics, clinical science, statistical genetics, and bioinformatics to develop a robust understanding of the cumulative stress burden for immigrants living in two Midwestern US cities including the association with disease and health status.
- Dr. Mersha (PI), a human geneticist who specializes in the genetics and epigenetics of complex diseases, is a native of Ethiopia. He has served as PI on previous R01, R03, and K-level funding, with over 100 publications. He is a founding Member of the Alliance for Research, Innovation, and Education, an international team of volunteer educators, researchers, and innovators established for partnership and to support Ethiopian universities and research centers to improve standards in education, research, innovation, and development capabilities through sharing knowledge and experience. Dr. Vaughn is a social psychologist with expertise in community-engaged research, mixed methods research, patient/family engagement, and health equity among immigrant populations. Dr. Vaughn and Dr. Mersha have an established partnership with many products of collaboration. Collaborative partnership with Cincinnati Children's Hospital Medical Center's Genetic Variation and Gene Discovery Core Facility, which will be the site of the DNA, RNA extraction, and epigenome work and bioinformatics analysis under the MPI's leadership.
- The research team has an established community partnership with two Ethiopian-serving agencies and a track record in conducting stress and epigenetic studies.

Weaknesses

- No major weaknesses.

3. Innovation:

Strengths

- The study team is investigating multiple ways in which stress becomes embodied in Ethiopian American immigrants; the incorporation of DNA methylation is a novel approach with this population.
- Weighted co-methylation network analysis will be used instead of single CpG site analysis to comprehensively capture epigenetic pathways between stress and health among EAIs.
- The team proposes to establish long-term community-academic partnerships with the EAI communities in both cities. This is built upon existing collaborative relationships. The team plans to build upon these relationships to conduct, culturally-relevant and contextually-grounded future intervention studies.

- Feasibility will be enhanced by the involvement of two EAI-serving community sites will serve as community partners for this project: 1) Mahdereselam Kidanemihret Orthodox Church, which provides services to Ethiopian immigrants living in the greater Cincinnati metro area. 2) Ethiopian Tewahedo Social Services, which facilitates culturally sensitive education, training, support services, and self-development opportunities for immigrants and refugees with emphasis on EAIs in the Columbus metro area.

Weaknesses

- No major weaknesses.

4. Approach:

Strengths

- Approximately 150 participants will be recruited (75 participants from each of the two sites; Cincinnati and Columbus). The sample size of 150 will provide 80% power to detect significant differences.
- Participant screening and enrollment will occur over 12 months.
- Well-described methods for data collection and analysis.
- Objective and subjective measures of stress/burden will be used, including measures that can be adapted to EAI participants.
- Missing data plan includes use of maximum likelihood estimation method or multiple imputation.
- This research and the novel approaches to measuring DNA methylation will contribute to knowledge about the health of U.S. populations beyond the EAI population that is the focus of this proposed study.

Weaknesses

- Are there stress measures that have been specifically validated among EAI that could be included in this proposed study?
- If one of the major study questions is how do we capture and define cumulative stress burden for immigrants, would it be helpful to collect qualitative data about the stress experiences of EAIs for future research studies?

5. Environment:

Strengths

- The research environment in Ohio is strong for this proposed study. The study builds on strong existing relationships with the community (including a community advisory board) and the research center.
- Strengths include Cincinnati Children’s Hospital Medical Center’s Genetic Variation and Gene Discovery Core Facility, the Center for Clinical and Translational Science and Training, Dr. Mersha’s robust laboratory space is also a strength.

Weaknesses

- No major concerns.

Study Timeline:

Strengths

- None noted by reviewer

Weaknesses

- A study timeline is referred to as being located in section 2.7, but I did not find it in the proposal documents.

Protections for Human Subjects:

Acceptable Risks and/or Adequate Protections

- No major concerns. Resources should be provided for mental health as the questionnaires could cause stress to the participants.

Data and Safety Monitoring Plan (Applicable for Clinical Trials Only):

Not Applicable (No Clinical Trials)

Inclusion Plans:

- Sex/Gender: Distribution justified scientifically
- Race/Ethnicity: Distribution justified scientifically
- For NIH-Defined Phase III trials, Plans for valid design and analysis: Not applicable
- Inclusion/Exclusion Based on Age: Distribution justified scientifically
- No major concerns.

Vertebrate Animals:

Not Applicable (No Vertebrate Animals)

Biohazards:

Not Applicable (No Biohazards)

Resource Sharing Plans:

Not Applicable (No Relevant Resources)

Authentication of Key Biological and/or Chemical Resources:

Acceptable

Budget and Period of Support:

Recommend as Requested

CRITIQUE 2

Significance: 2

Investigator(s): 4
Innovation: 3
Approach: 5
Environment: 1

Overall Impact: This R21 application seeks to measure and define stress burden (aim 1) among Ethiopian American and assess whether stress burden is associated with DNAm (aim 2). To support these goals, this proposal seeks to recruit a sample of 150 Ethiopian American immigrants from 2 sites (Columbus and Cincinnati Ohio) to assess DNAm, stress burden (via self-report) and physical health outcomes (hypertension, blood glucose, BMI). Score driving strengths in the approach and innovation include genomic research among a homogenous immigrant group and an underresearched population, the use of data dimensionality reduction for DNAm data, the consideration of DNAm in the context of immigrant stress. The study team has extensive expertise in epigenetics and building immigrant community-based partnerships. Score driving weaknesses include the practical study administration concern that there is sufficient staff effort to recruit these samples, the lack of mental health assessments, the lack of expertise in stress on the study team. Overall, this represents a highly significant and innovative application by a strong investigative team and environment that is dampened by the major concerns outlined above.

1. Significance:

Strengths

- Understanding stress burden among immigrants and those of Ethiopian ancestry is important and remains largely uninvestigated.

Weaknesses

- There are limited outcomes studied (see approach comments) which may diminish the informativeness of this work for health outcomes.

2. Investigator(s):

Strengths

- MPI Mersha has expertise in epigenetics of complex disease.
- MPI Vaughn has expertise working with immigrant populations and developing community-based participatory research programs.
- Co-I Ding provides analytic support.
- There is established collaboration between MPIs and between MPI Mersha and Co-I Ding.
- Well specified MPI plan and clear delineation of responsibilities.

Weaknesses

- There does not appear to be expertise in stress among the study team.

3. Innovation:

Strengths

- Collecting epigenetic data in a homogenous immigrant population within the US is novel and would contribute to ongoing efforts to further diversify genetic research.

- Weighted co-methylation network analyses are useful for epigenetic data dimensionality reduction.

Weaknesses

- There is a lack of other DNAm measures (e.g., aging/clock)
- Limiting outcomes to physical health conditions limits innovation.
- Stress has been well studied in the context of DNAm.

4. Approach:

Strengths

- Interdisciplinary approach evaluating putative biological mechanisms associated with immigrant stress.
- Connections to community partner sites (Mahdereselam Kidanemihret Orthodox Church & Ethiopian Tewahedo Services) with letters of support.
- Collecting epigenetic data in a homogenous immigrant population.
- Measurement of blood pressure and A1c, and BMI.
- Careful consideration of covariates in epigenetic analyses – e.g., batch, cell type, ancestry.

Weaknesses

- There does not appear to be sufficient staff to support data collection (see budget comments).
- It is not clear that outcomes assessed adequately capture stress related conditions. The grant is contextualized with physical and mental health conditions but there is no assessment of mental health conditions present. Outcomes measures are restricted to physical health and chronic conditions – e.g., obesity, diabetes, hypertension/high blood pressure. Depending on the age of the sample, there may not be sufficient variability in some of these.
- Identifying genetic modules is a nice data dimensionality reduction technique – however, given the focus on stress-related conditions and their associations with age, the lack of other data dimensionality reduction techniques for DNAm such as epigenetic clocks/aging signatures, is unclear.
- Covariates include age, immigration duration, and sex. There is no assessment of prior stress exposure (e.g., before immigrating) outside of the past 5 years, which would appear to be an important consideration for assessing stress burden among immigrants. Consideration of stress buffering aspects – e.g., SES would seem to be a further important variable to consider in addition to the presence of financial strain in the Boen measure (e.g., stressors may be less burdensome if one has means that help address them) or social support are not considered.
- Latent profile analysis. It is unclear what will be fed into the latent profile analysis? Would this be the 5 scales from the stress of immigration survey (language, immigrant status, work issues, yearning for family and home country, cultural dissonance), 2 Boen measures (number of stressors, composite total exposure) and the perceived stress scale total? There are no alternatives if latent profile analyses do not generate distinct groups or generate adequately sized groups.
- How the SOIS would be adapted for Ethiopian American immigrants is unclear.

- There is some evidence that the PSS may represent trait neuroticism and reflect trait like reactivity to stress, at least in some populations. Its use alongside other indices of stress exposure is unclear and may introduce heterogeneity into the concept of stress burden.
- (non-score driving) The proposal notes transmission of epigenetic signatures across generations highlighting the importance of DNAm as a contributor to health outcomes. Future research in this sample may benefit from an assessment of participants about the presence of offspring and willingness for them to participate.
- It is unclear where expected effect sizes used in the power analysis were derived from.
- It is unclear how multiple testing will be adjusted for.

5. Environment:

Strengths

- Connections to community partner sites with letters of support.

Weaknesses

- Unclear that there is sufficient staff time for recruitment and data collection (see comments in budget).

Protections for Human Subjects:

Acceptable Risks and/or Adequate Protections

Data and Safety Monitoring Plan (Applicable for Clinical Trials Only):

Not Applicable (No Clinical Trials)

Inclusion Plans:

- Sex/Gender: Distribution justified scientifically
- Race/Ethnicity: Distribution justified scientifically
- For NIH-Defined Phase III trials, Plans for valid design and analysis:
- Inclusion/Exclusion Based on Age: Distribution justified scientifically
- Restricted race is well justified.

Vertebrate Animals:

Not Applicable (No Vertebrate Animals)

Biohazards:

Not Applicable (No Biohazards)

Resubmission:

- n/a

Renewal:

- n/a

Revision:

- n/a

Resource Sharing Plans:

Acceptable

- Planned deposit of genetic data consistent with NIH policy.

Authentication of Key Biological and/or Chemical Resources:

Not Applicable (No Relevant Resources)

Budget and Period of Support:

Recommended budget modifications or possible overlap identified:

- The budget justification notes that 2 community coordinators would be hired (1 for each site) who would be responsible for recruitment, retention, data collection, interpretation, and dissemination. They will be paid $31/hour each and the budget in year 1 for this will not exceed $9,660. This would equate to ~300 total hours for both community coordinators (~150 each or ~ 1 month of work). This would appear to be insufficient to recruit and retain 150 individuals at each site and collect data from them and coordinate with investigators and community partners.

CRITIQUE 3
Significance: 4
Investigator(s): 2
Innovation: 4
Approach: 6
Environment: 1

Overall Impact: This R21 application aims to identify stress burden profiles among 150 Ethiopian American immigrants (EAIs) and explore the association between their psychosocial stress and DNA methylation. However, there are several issues with the conceptual framework and methodology of the study. First, the study lacks a comprehensive multi-level conceptual model that illustrates the various sources of stress and the epigenomic mechanisms involved. This framework is essential for understanding the relationship between stress and DNA changes among EAIs. Second, the application does not adequately present the stress and health problems experienced by EPIs to justify the significant health disparities observed in this population. Currently, it is unclear how EAIs' stress burden profiles (Aim 1) are important for explaining epigenetic changes and reducing health disparities (Aim 2). Third, the specific psychological and environmental stressors that have caused epigenetic changes in humans remain unknown, as individuals are typically exposed to multiple factors simultaneously. The applicants should provide a better justification for how they intend to disentangle the DNA changes associated with stress from other factors such as lifestyle, substance use, and genetic inheritance. Furthermore, the study design lacks a control group for comparison with the 150 EAIs, which is necessary to empirically attribute the observed epigenetic changes to immigration stress and its

influence on increased risk for chronic diseases. The use of cross-sectional, self-reported surveys to define cumulative or chronic stress burden is also less valid compared to employing repeated measures. Moreover, it is unclear how the surveys will be culturally appropriate for EAIs, as they have not been validated and tested within this specific population. In addition, the enrollment criteria and screening instruments for selecting distressed EAIs are not clearly defined. While the measurement of DNA methylation is sound and specified, the details regarding how and when saliva samples will be collected are lacking. It is also unknown how saliva cell types will be identified and analyzed. The assumption of DNAm modules as mediators lacks citations to support its validity. Moreover, the links between cultural ties, social and environmental stress, and DNA changes are not clearly explained, limiting their ability to inform future research directions. Considering these conceptual and methodological concerns, the knowledge gained from this application is relatively limited.

Study Timeline:

Strengths

- The tasks for the first six months, including personnel hiring and training, community advisory board meetings, are acceptable.

Weaknesses

- Timeline for the feasibility of enrolling 150 distressed patients is not justified.

Protections for Human Subjects:

Acceptable Risks and/or Adequate Protections

Data and Safety Monitoring Plan (Applicable for Clinical Trials Only):

Acceptable

Inclusion Plans:

- Sex/Gender: Distribution justified scientifically
- Race/Ethnicity: Distribution justified scientifically
- For NIH-Defined Phase III trials, Plans for valid design and analysis: Not applicable
- Inclusion/Exclusion Based on Age: Distribution justified scientifically

Vertebrate Animals:

Not Applicable (No Vertebrate Animals)

Biohazards:

Not Applicable (No Biohazards)

Resource Sharing Plans:

Acceptable

Authentication of Key Biological and/or Chemical Resources:

Not Applicable (No Relevant Resources)

Budget and Period of Support:
Recommend as Requested

THE FOLLOWING SECTIONS WERE PREPARED BY THE SCIENTIFIC REVIEW OFFICER TO SUMMARIZE THE OUTCOME OF DISCUSSIONS OF THE REVIEW COMMITTEE, OR REVIEWERS' WRITTEN CRITIQUES, ON THE FOLLOWING ISSUES:

PROTECTION OF HUMAN SUBJECTS: ACCEPTABLE

INCLUSION OF WOMEN PLAN: ACCEPTABLE

INCLUSION OF MINORITIES PLAN: ACCEPTABLE

INCLUSION ACROSS THE LIFESPAN: ACCEPTABLE

COMMITTEE BUDGET RECOMMENDATIONS: The coordinators at the respective sites may be underfunded and may have insufficient time to complete all the work and it seems they may not be traveling between sites, adding to the workload at each site that cannot be shared.

Footnotes for 1R21MD019192-01; PI Name: Mersha, Tesfaye B.

+ Derived from the range of percentile values calculated for the study section that reviewed this application.

NIH has modified its policy regarding the receipt of resubmissions (amended applications).See Guide Notice NOT-OD-18-197 at https://grants.nih.gov/grants/guide/notice-files/NOT-OD-18-197.html. The impact/priority score is calculated after discussion of an application by averaging the overall scores (1-9) given by all voting reviewers on the committee and multiplying by 10. The criterion scores are submitted prior to the meeting by the individual reviewers assigned to an application, and are not discussed specifically at the review meeting or calculated into the overall impact score. Some applications also receive a percentile ranking. For details on the review process, see http://grants.nih.gov/grants/peer_review_process.htm#scoring.

MEETING ROSTER

Biobehavioral Mechanisms of Emotion, Stress and Health Study Section
Biobehavioral and Behavioral Processes Integrated Review Group
CENTER FOR SCIENTIFIC REVIEW
MESH
06/28/2023 - 06/29/2023

Notice of NIH Policy to All Applicants: Meeting rosters are provided for information purposes only. Applicant investigators and institutional officials must not communicate directly with study section members about an application before or after the review. Failure to observe this policy will create a serious breach of integrity in the peer review process, and may lead to actions outlined in NOT-OD-22-044 at https://grants.nih.gov/grants/guide/notice-files/NOT-OD-22-044.html, including removal of the application from immediate review.

CHAIRPERSON(S)

MONK, CATHERINE E, PHD
PROFESSOR
DEPARTMENT OF OBSTETRICS AND GYNECOLOGY
AND PSYCHIATRY
COLLEGE OF PHYSICIANS AND SURGEONS
COLUMBIA UNIVERSITY MEDICAL CENTER
NEW YORK, NY 10032

MEMBERS

BAKER, FIONA C, PHD
SENIOR PROGRAM DIRECTOR
CENTER FOR HEALTH SCIENCES
SRI INTERNATIONAL
MENLO PARK, CA 94025

BLACK, DAVID SCOTT, PHD *
ASSOCIATE PROFESSOR
DEPARTMENT OF POPULATION AND PUBLIC HEALTH
SCIENCE
KECK SCHOOL OF MEDICINE
UNIVERSITY OF SOUTHERN CALIFORNIA
LOS ANGELES, CA 90033

BOGDAN, RYAN H, PHD
ASSOCIATE PROFESSOR
DEPARTMENT OF PSYCHOLOGICAL
AND BRAIN SCIENCES
WASHINGTON UNIVERSITY IN ST. LOUIS
ST. LOUIS, MO 63130

BOGGERO, IAN ANDRES, PHD *
ASSISTANT PROFESSOR
DEPARTMENTS OF ORAL HEALTH SCIENCE AND
PSYCHOLOGY
UNIVERSITY OF KENTUCKY
LEXINGTON, KY 40506

BRENNAN, PATRICIA A, PHD
PROFESSOR
DEPARTMENT OF PSYCHOLOGY
EMORY UNIVERSITY
ATLANTA, GA 30322

CARTER, JASON R, PHD
DEAN AND PROFESSOR
ROBBINS COLLEGE OF HEALTH AND HUMAN SCIENCES
BAYLOR UNIVERSITY
WACO, TX 76798

CHIDAMBARAN, VIDYA, MD
PROFESSOR
DEPARTMENT OF ANESTHESIA
CINCINNATI CHILDREN'S HOSPITAL
CINCINNATI, OH 45229

CONRAD, AMY LYNN, PHD *
ASSOCIATE PROFESSOR
THE STEAD FAMILY DEPARTMENT OF PEDIATRICS
DEVELOPMENTAL AND BEHAVIORAL PEDIATRICS DIVISION
UNIVERSITY OF IOWA CHILDREN'S HOSPITAL
IOWA CITY, IA 52242

DONG, YANBIN, PHD, MD
PROFESSOR
DEPARTMENT OF MEDICINE
MEDICAL COLLEGE OF GEORGIA
AUGUSTA UNIVERSITY
AUGUSTA, GA 30912

GEHRMAN, PHILIP RICHARD, PHD
PROFESSOR
DEPARTMENT OF PSYCHIATRY
PENN SLEEP CENTER
UNIVERSITY OF PENNSYLVANIA
PHILDELPHIA, PA 19104

GISCOMBE, CHERYL L, PHD
PROFESSOR
SCHOOL OF NURSING
UNIVERSITY OF NORTH CAROLINA AT CHAPEL HILL
CHAPEL HILL, NC 27599

GORKA, STEPHANIE, PHD *
ASSOCIATE PROFESSOR
DEPARTMENT FOR PSYCHIATRY AND BEHAVIORAL HEALTH
THE OHIO STATE UNIVERSITY
COLUMBUS, OH 43210

LARSON, CHRISTINE L, PHD
PROFESSOR
DEPARTMENT OF PSYCHOLOGY
UNIVERSITY OF WISCONSIN-MILWAUKEE
MILWAUKEE, WI 53211

LAURENT, HEIDEMARIE KAISER, PHD
ASSOCIATE PROFESSOR
DEPARTMENT OF HUMAN DEVELOPMENT AND FAMILY STUDIES
THE PENNSYLVANIA STATE UNIVERSITY
UNIVERSITY PARK, PA 16802

LEVENDOSKY, ALYTIA A, PHD
PROFESSOR
DEPARTMENT OF PSYCHOLOGY
MICHIGAN STATE UNIVERSITY
EAST LANSING, MI 48824

LEYRO, TERESA MARIA, PHD *
ASSOCIATE PROFESSOR
DEPARTMENT OF PSYCHOLOGY
RUTGERS, THE STATE UNIVERSITY OF NEW JERSEY
PISCATAWAY, NJ 08854

LOPEZ, JUAN F, MD *
ASSOCIATE PROFESSOR EMERITUS
DEPARTMENT OF PSYCHIATRY
UNIVERSITY OF MICHIGAN MEDICAL CENTER
ANN ARBOR, MI 48109

MURROUGH, JAMES WARREN, MD, PHD
PROFESSOR
DEPARTMENT OF PSYCHIATRY AND NEUROSCIENCE
ICAHN SCHOOL OF MEDICINE AT MOUNT SINAI
NEW YORK, NY 10029

PRATHER, ARIC ANDREW, PHD
PROFESSOR
DEPARTMENT OF PSYCHIATRY
SCHOOL OF MEDICINE
UNIVERSITY OF CALIFORNIA SAN FRANCISCO
SAN FRANCISCO, CA 94118

SALMOIRAGO-BLOTCHER, ELENA, MD, PHD
ASSOCIATE PROFESSOR
DEPARTMENT OF MEDICINE
AND PSYCHIATRY AND HUMAN BEHAVIOR
BROWN UNIVERSITY SCHOOL OF MEDICINE
PROVIDENCE, RI 02903

SMITH, MICHAEL T, PHD
PROFESSOR
DEPARTMENT OF PSYCHIATRY AND BEHAVIORAL SCIENCES
BAYVIEW MEDICAL CENTER
SCHOOL OF MEDICINE
JOHNS HOPKINS UNIVERSITY
BALTIMORE, MD 21224

SULLIVAN, ELINOR L, PHD
PROFESSOR
DEPARTMENT OF PSYCHIATRY
OREGON HEALTH AND SCIENCE UNIVERSITY
PORTLAND, OR 97239

WANG, JUDY HUEI-YU, PHD *
ASSOCIATE PROFESSOR
DEPARTMENT OF ONCOLOGY
GEORGETOWN UNIVERSITY MEDICAL CENTER
WASHINGTON, DC 20007

WEIERICH, MARIANN R, PHD
PROFESSOR
DEPARTMENT OF PSYCHOLOGY
UNIVERSITY OF NEVADA
RENO, NV 89557

YIP, TIFFANY, PHD
PROFESSOR
DEPARTMENT OF PSYCHOLOGY
FORDHAM UNIVERSITY
BRONX, NY 10458

ZHEN-DUAN, JENNY, PHD *
ASSISTANT PROFESSOR
DEPARTMENTS OF MEDICINE AND PSYCHIATRY
INSTITUTE MASSACHUSETTS GENERAL HOSPITAL AND HARVARD MEDICAL SCHOOL
BOSTON, MA 02114

SCIENTIFIC REVIEW OFFICER

MASON-MAH, BRITTANY L., PHD
SCIENTIFIC REVIEW OFFICER
CENTER FOR SCIENTIFIC REVIEW
NATIONAL INSTITUTES OF HEALTH
BETHESDA, MD 20892

* Temporary Member. For grant applications, temporary members may participate in the entire meeting or may review only selected applications as needed.

Consultants are required to absent themselves from the room during the review of any application if their presence would constitute or appear to constitute a conflict of interest.
